# Ultrasensitive Quantification of Cytokine Proteins in Single Lymphocytes From Human Blood Following *ex-vivo* Stimulation

**DOI:** 10.3389/fimmu.2018.02462

**Published:** 2018-10-23

**Authors:** Ankit Saxena, Pradeep K. Dagur, Alisha Desai, John Philip McCoy

**Affiliations:** National Heart, Lung, and Blood Institute, National Institutes of Health, Bethesda, MD, United States

**Keywords:** IFN-γ, TNF-α, T cell, SiMoA, flow cytometry

## Abstract

In this study we demonstrate the feasibility of direct, quantitative measurement of cytokine proteins in single human CD8 lymphocytes from fresh peripheral blood of healthy donors following a brief *ex vivo* stimulation. Cytokine-secreting cells were identified using cell surface “catch” reagents and single cell data were obtained by sorting of individual cytokine-secreting cells into 96 well plates containing lysis buffer followed by analysis using ultrasensitive immunoassays for interferon gamma (IFN-γ) and tumor necrosis factor alpha (TNF-α). CD8 cells negative for cytokine production, as determined by the cell surface catch reagents were used as negative controls. Furthermore, studies were undertaken to compare the mean fluorescence intensity (MFI) values of cytokine staining by flow cytometry with the quantification of cytokines using the current method. This study demonstrates that it is feasible to quantify cytokines from individual primary cells. A shift from qualitative to quantitative determinations of cytokine protein levels in single cells will permit more precise and reproducible studies of heterogeneity in the immune system and can be accomplished with readily available instrumentation.

## Introduction

The increasing awareness of cellular heterogeneity among cell populations that might appear uniform by morphologic or phenotypic characteristics has prompted techniques for analysis of single cells (1, 2). This heterogeneity has been observed using technologies such as flow and mass cytometry (3, 4), however, the study of single cell isolates from the heterogenous populations has involved primarily genomic and transcriptomic approaches. Data related to this heterogeneity on the protein level, particularly involving quantification of proteins, has been extremely limited. Most immunoassays used to study cellular proteins, such as ELISA, Luminex bead arrays, and Western blots, have sensitivities in the picogram to high nanogram per ml range which are too insensitive to detect and quantify proteins from individual cells and therefore require quantification of analytes to be performed on large number of cells which are assumed to be homogeneous. These conventional methods are accessible and simple but can incorrectly describe the distribution of behavior among individual cells in the sample. This is important because even genetically identical cells are dynamic and varied in their responses to stimuli due to epigenetic differences, the stochastic nature of intracellular signaling, and the small number of molecules which are engaged (5, 6). Technologies that are excellent at detection of protein on individual cells, such as flow and mass cytometry, do not collect data that yield precise quantification of protein amounts but rather describe relative protein levels as “bright” or “dim.”

Studying single cells has been a challenge so far and poses limitations for studying crucial changes in cellular functions during diseases pathogenesis and homeostasis. There have been numerous single cell studies measuring mRNA as a surrogate for the proteome (7, 8). Results from such studies has often been debated and it has been shown that the amount of mRNA dose not correlate with the amount of protein expressed (9, 10). Therefore, it is more important to characterize protein expression at single cell level. Previous studies to quantify cellular proteins at the single cell level have been reported (11–14). Several of these require custom devices which are not commercially available (11, 12). Others require the introduction of a fluorescent protein reporter or fluorogenic substrates to achieve single cells quantification of proteins, which would preclude the study of fresh primary cells isolates (13, 14). SiMoA—a novel ultrasensitive immunoassay that makes use of arrays of femtoliter-sized reaction chambers has recently been introduced as a commercial product (15, 16). This new technology, termed SiMoA (Single Molecule Array) enables measurement of proteins in a variety of different mediums at attomolar concentrations. Unlike ELISA (enzyme-linked immunosorbent assay), an analog system, SiMoA does not require large volumes that dilute reaction product. Array volumes are about 2 billion times smaller than a conventional ELISA. Thus, if a labeled protein is present, a rapid buildup of fluorescent product is generated. One recent study used the SiMoA technology to detect prostate-specific antigen (PSA) in cultured LNCaP cell lines expressing different amounts of PSA (17). That study could detect quantifiable differences in the amount of PSA between single cells isolated from the high- and low- PSA expressing cell lines and report the number of PSA molecules measured in each cell. Unfortunately, this study did not examine PSA-negative cells as a true negative control and examined a cell line grown *in vitro* rather than primary cells.

Heterogeneity in immune cell populations allows flexibility, particularly during dynamic processes such as differentiation and antigenic response and the study of this heterogeneity is a challenge that is meaningfully addressed by single cell analysis (18, 19). Cytokines are pivotal in development of functional heterogeneity among T cell subsets. They are small proteins that are important in cell signaling, effector function and communication. Quantifying these proteins at the single cell level will enable a better understanding of cellular pathways and behavior using measurements that are absolute rather than relative.

Based on the paucity of available techniques to quantify the amount of a particular protein in single cells using readily available instrumentation, and the promising study of PSA using the SiMoA, we sought to determine if this technology could be adapted to quantify intracellular cytokines in lymphocytes. We report here the ultrasensitive quantification of major pro-inflammatory cytokines like TNF-α and IFN-γ in freshly isolated single human T cells.

## Materials and methods

**Figure 1:**
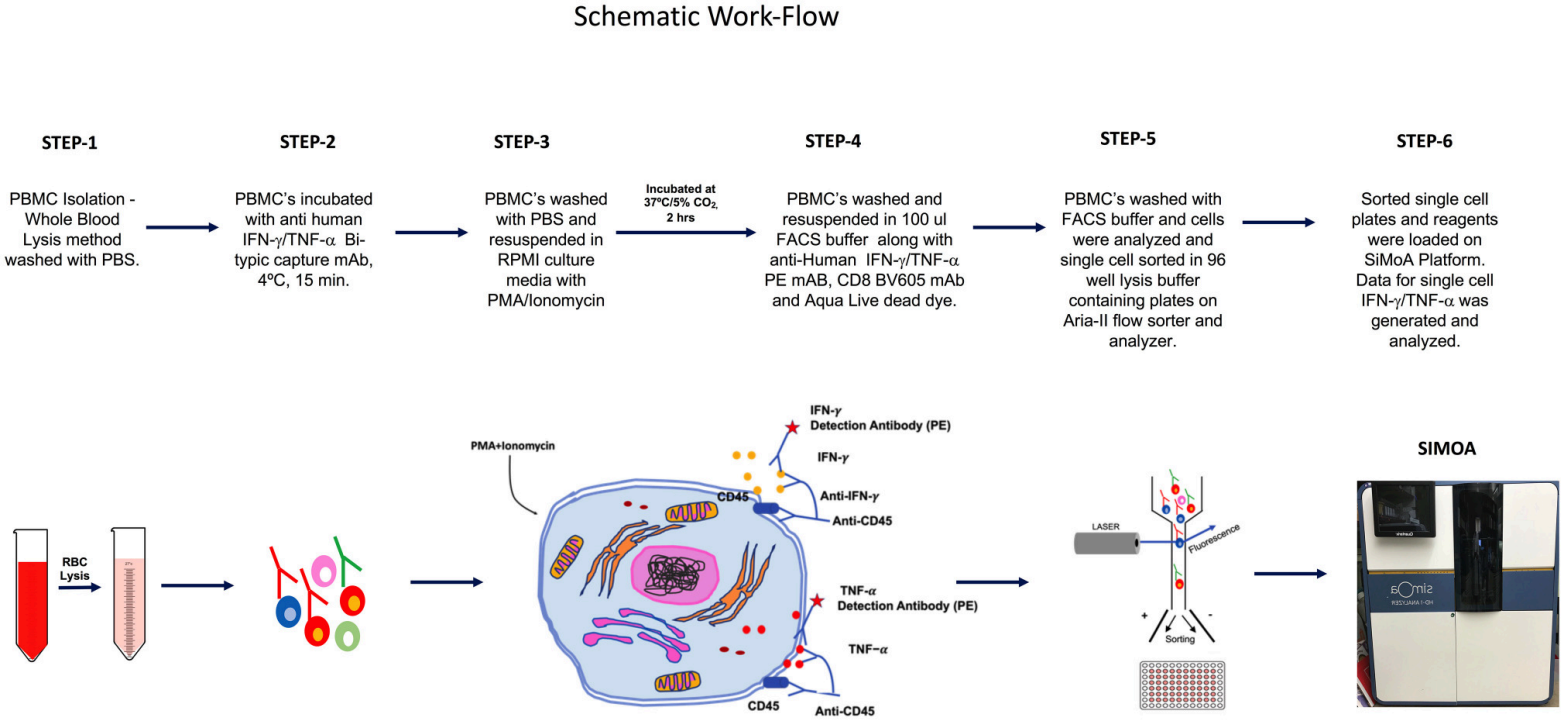
SiMoA schematic workflow showing stepwise procedures performed to quantify cytokines in single cells.

The overall schematic of the workflow for this study is shown in Figure 1.

### Healthy donors

Human peripheral blood mononuclear cells (PBMC) was collected in sodium heparin vacutainers [Becton Dickinson (BD), San Jose, CA] from healthy donors at National Institutes of Health, Clinical Center. The samples were collected after approval by the Institutional Review Board and signed written informed consent by donors (protocol-07-H-0113).

### Reagents

The SiMoA HD-1 analyzer, SiMoA consumables, and IFN-γ (SiMoA™ IFN-γ,138 Kit) and TNF-α (SiMoA™ TNF-α 2.0, 208 Kit) were purchased from Quanterix, Lexington, MA. IFN-γ and TNF-α secretion assay detection kits (PE conjugated) were purchased from Miltenyi Biotech, Auburn, CA. Anti-human CD8 (BV 605, clone-SK1) was obtained from BD Biosciences and Live/Dead Fixable Aqua (ThermoFisher Invitrogen, Grand Island, NY). RPMI-1640 (ThermoFisher Gibco, Grand Island, NY) supplemented with 10%FCS and 1X antimycotic and antibiotic solution were used for culture. FACS staining buffer (1X PBS, 0.5% bovine serum albumin, 0.025 mM EDTA) were used for FACS staining. The lysis buffer consisted of lysis Buffer 17 (R&D Systems) and Halt™ Protease Inhibitor Cocktail (Thermo Fisher Scientific, Rockford, IL).

### Cell culture and stimulation

All samples were processed within 24 h of draw. Whole blood pellets were re-suspended in ACK lysing buffer (Quality Biologicals, Gaithersburg, MD), and incubated for 2–3 min at room temperature to lyse RBC and then washed with PBS by centrifugation. PBMC yield and viability were determined using trypan blue dye and cell counting was performed with hemocytometer.

### IFN-γ and TNF-α capture assay using capture antibodies

IFN-γ and TNF-α -secreting cells were detected using the secretion assay kits (Miltenyi Biotec Inc. Auburn, CA) according to the manufacturer's instructions. Briefly, 2–3 × 10^6^ PBMC were stimulated with Phorbol 12-myristate 13-acetate (PMA, 10 ng/ml; Sigma-Aldrich, St. Louis, MO) and ionomycin (500 ng/ml Sigma-Aldrich, St. Louis, MO) for 3 h at 37°C, 5% CO_2_. Cell were washed once with cold PBS. Cell pellet was suspended in 80 μl cold medium and 20 μl IFN-γ or TNF-α catch reagent (a bi-specific antibody reagent directed against CD45 and to either IFN-γ or TNF-α). After 10 min of incubation (labeling) at 4°C, 1 ml of warm (37°C) medium was added. The cells were placed at 37°C on a slow rotating platform to allow cytokine secretion for 45 min. The cells were immediately placed on ice and then washed with cold buffer (300 g, 10 min, 4°C) and re-suspended in 80 μl cold buffer. The secreted IFN-γ, bound to the catch reagent, was stained with 20 μl PE-conjugated IFN-γ or TNF-α specific antibody (Detection Reagent). After an incubation period of 10 min at 4°C, the cells were washed with cold buffer, spun down (300 g, 10 min, 4°C) and re-suspended in FACS-buffer. The cells were stained for expression of surface marker CD8 and Live/Dead Fixable Aqua (Life Technologies, Grand Island, NY).

### TNF-α capture assay using TNF-α processing inhibitor (TAPI-0)

PBMC were resuspended at 10^6^ cells/ml in RPMI, sterile filtered prior to use. Cells were then stimulated with PMA (10 ng/ml; Sigma-Aldrich) Ionomycin (500 ng/ml Sigma-Aldrich) in the presence of 15 μl of anti-TNF-α PE (BD Biosciences) and 10 μM of TAPI-0 (Calbiochem, Burlington, MA) for 3 h at 37°C, 5% CO_2_. Following the 3 h incubation period with anti-TNF-α PE mAb, cells were not re-stained with anti-TNF-α in any subsequent steps. Following incubation, cells were stained with surface markers CD8 and Live/Dead Fixable Aqua (Life Technologies, Grand Island, NY).

### Cell sorting

The stained cells were sorted into CD8+ sIFN-γ-bright and CD8+ sIFN-γ-negative or CD8+ sTNF-α-bright and CD8 + sTNF-α-negative subpopulations. Isolated single cells were sorted in 96 well V bottom plate (Quanterix Corporation) containing 25 ul of lysis Buffer 17 (R&D Systems) and Halt™ Protease Inhibitor Cocktail (Thermo Fisher Scientific, Rockford, IL). A FACSAria SORP™ sorter equipped with 355, 405, 488, 532, and 638 nm laser lines and DIVA™ 8.0.1 software (BD) was used for sorting. To validate that a single CD8+ cell is present per well we performed some additional sorts where CFSE (0.5 uM) (Invitrogen, Oregon) stained CD8+ T cells were sorted in 50 μl of RPMI-1640 (ThermoFisher Gibco, Grand Island, NY) in 96 well flat bottom plate (Costar, Corning Inc., NY). Single cell per well were observed and counted using Nikon eclipse TS100 inverted microscope (Melville, NY). Sorting 2 plates this way (192 wells) we found that 190 wells contained 1 cell, 2 contained no cells, 0 contained 2 or more cells, resulting in a 99% accurate deposition of single cells.

### SiMoA assay validation procedure

The standard curves for both IFN-γ and TNF-α assay kits were readjusted to address lower concentration range of the single cell assay. The manufacturers protocol defined standard curve was modified by dropping top 3 standard curve points for single cell IFN-γ assay and introducing three curve points at the lower end. For TNF-α we dropped top 2 standard curve points and added 2 points at the lower end of the curve. Sample diluent was prepared by adding lysis Buffer 17 (R&D Systems) and SiMoA assay kit sample diluent (at ratio 1: 5.4). Triplicate measurements of serially diluted samples, spiked with recombinant IFN-γ and TNF-α protein, are tested on a minimum of 3 runs. For each run a calibration curve is established and the concentration and CV% of each sample dilution determined. A power curve is fit to a plot of concentration (x-axis) vs. CV% (y-axis), to which LLoQ is the lowest concentration with ≤20% CV (Supplementary Figures 1A,B) calculated from solving the power equation.

### Single molecule array (SiMoA) analysis

Isolated single cells were collected in 96 well V bottom plate (Quanterix Corporation) containing 25 ul of lysis Buffer 17 (R&D Systems) as described above. Cells were finally diluted in 110 μl of sample diluent provided in SiMoA kit to obtain a total sample volume of 135 μL per single cell. All cell samples and assay reagents (capture beads, detection antibody, SBG, and RGP) were loaded into the appropriate reagent bays in the HD-1 Analyzer. The average number of enzymes per bead (AEB) was calculated, based on the number of active wells and the total number of beads, using Poisson statistics and the digital or analog methods previously described (20). IFN-γ and TNF-α cytokine calibration curves were fit with a four-parameter logistic weighted regression to determine protein concentrations in the samples from measured AEB values. The number of molecules in a single cell were calculated by multiplying Avogadro's constant by the number of Moles in the sample. The number of Moles was calculated by dividing the weight of IFN-γ or TNF-α in gm (single cell) by the molecular weight of IFN-γ or TNF-α the molecular weights of IFN-γ and TNF-α were obtained from the UniProt Consortium. The lower limit of quantitation (LLoQ) values for IFN-γ and TNF-α were: IFN-γ−0.01379 pg/ml; and TNF-a−0.0028 pg/ml). The LLoQ was determining using the method of Andreasson et al. (21).

### IFN-γ competitive inhibition assay

CD8+ IFN-γ+ PMA stimulated positive and negative cells were sorted as described above. In a control 96 well plate, 5 and 10 cells per well (sIFN-γ pos and neg) were sorted in replicates of 10 wells containing 25 μl of lysis Buffer 17 (R&D Systems). Cells were finally diluted in 110 μl of assay diluent provided in SiMoA kit to obtain a total sample volume of 135 μL per well. A separate 96 well flat bottom plate was coated with anti-Human IFN-γ antibody (clone-MD-1, Bio-legend, San Diego, CA) to determine the specificity of this assay by inhibition of the IFN-γ measurements by SiMoA. Anti-Human IFN-γ antibody was coated using Coating Buffer—PBS (prepared using 137 mM NaCl, 2.7 mM KCl, 8.1 mM Na_2_HPO_4_, and 1.5 mM KH_2_PO_4_ in PBS, all from Sigma-Aldrich, MO, USA) overnight at 4°C followed by a wash with PBS before use. Diluted lysates, identical to those in the control plate, were incubated in the plate coated with IFN-γ antibody for 45 min and then transferred to SiMoA plates for final readout. Recombinant human IFN-γ protein provided with the Quanterix kit (SiMoA™ IFN-γ,138 Kit) was used as a control (at 7.4 pg/ml concentration) in replicates for the inhibition experiment.

### Statistics

Comparison between the cytokine positive and negative cells was performed using one-way ANOVA with Dunnett's multiple comparison post-test. We tested the association between sIFN-γ MFI values and cellular IFN-γ concentrations using Spearman correlation. Results from inhibition assay were analyzed by applying the Mann–Whitney test. All analyses were carried out using GraphPad Prism software (version 7.0, GraphPad software, San Diego, USA). The level of significance was set at *p* < 0.05. For statistical analyses, values below the LLoQ were assigned the value of the LLoQ.

## Results

### Cytokine capture assay

**Figure 2:**
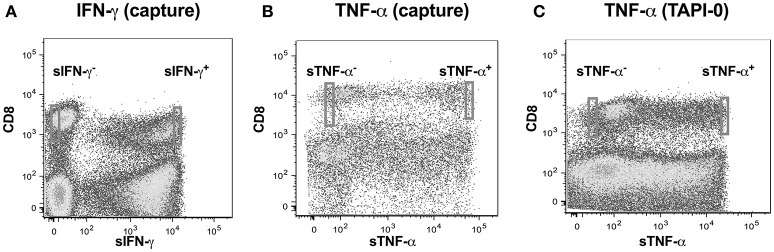
Cytokine capture assay for IFN-γ and TNF-α in CD8 T cells. Examples of gating strategies for sorting cells that were either IFN-γ and TNF-α positive. Cells were labeled with CD8 and **(A)** IFN-γ using a bispecific CD45/IFN-γ capture reagent; **(B)** TNF-α using a bispecific CD45/TNF-α capture reagent; or **(C)** TNF-α using a TNF-α capture assay with TNF-α processing inhibitor (TAPI-0). Stained cells were acquired and sorted on BD-FACS-Aria sorter.

We adopted a non-invasive cytokine capture assay technique for identifying cells secreting IFN-γ and TNF-α. We focused on CD8 T cells as PMA often results in significant downregulation of CD4 (22, 23) while CD8 expression on T cells is only marginally affected (24). A cytokine specific catch reagent which is an anti- IFN-γ or TNF-α monoclonal antibody (mouse IgG1) conjugated to CD45 specific monoclonal antibody (mouse IgG2a) is attached to the cell surface of all polyclonally stimulated leukocytes as described above. Cells are then washed to remove any excessive antibody and then incubated with PE conjugated anti-detection IFN-γ or TNF-α antibody and analyzed by flow cytometry. Our goal was to examine single cell concentrations of both IFN-γ or TNF-α as well as the linearity of the concentrations over a range of cell numbers. We thus sorted cell in following number order−1, 2, 5, and 10 from either sIFN-γ bright and sIFN-γ neg or TNF-α bright and TNF-α neg cells into 96 well plates (Figures 2A,B). For each cell number sorted we had 10 replicates per plate and experiments were repeated three times.

TNF-α production by CD8+ T-lymphocytes was also evaluated by another method where secreted TNF-α was captured by using a TNF-α processing inhibitor (TAPI-0) as reported earlier (25). Briefly, the process by which TNF-α is released from cell surface to become soluble TNF-α by the activity of TACE (TNF-α converting enzyme) is inhibited by TAPI-0. The fluorescence conjugated anti-TNF-α antibody in the culture binds to this surface TNF-α and is helpful in detecting cells actively secreting this cytokine (Figure 2C).

### Quantification of IFN-γ in single CD8 T cells

SiMoA was employed to quantify IFN-γ in single CD8 T cells. The estimation of the approximate detectable number of molecules in 135 μl of assay diluent is derived from single cells collected from brightest and dimmest gated IFN-γ producing cells based on their fluorescent intensity in the channel of detection.

**Figure 3:**
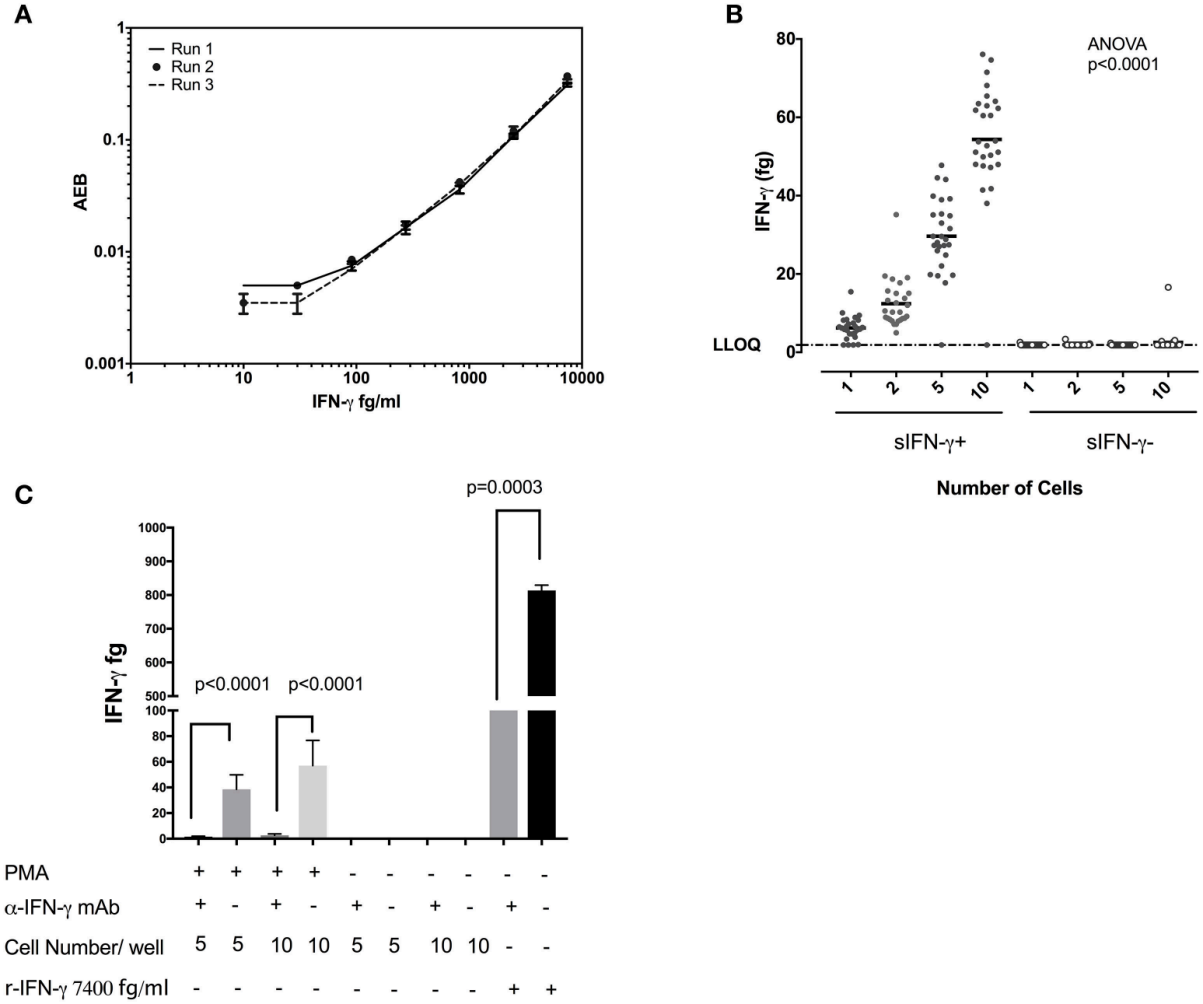
Quantification of IFN-γ in single CD8 T cells. **(A)** Reproducibility of the SiMoA standard curves for IFN-γ. Standard concentrations of IFN-γ were used to generate curves to calculate the unknown concentrations in cells **(B)** Comparison of measured IFN-γ protein at 1, 2, 5, and10 cell levels in CD8 cells. The amount of IFN-γ measured in femtograms of lysates from cell surface IFN-γ-positive cells or cells that were cell surface IFN-γ-negative. Left are data from IFN-γ-positive cells, right are data from IFN-γ-negative cells. Horizontal axis is the number of cells sorted into each lysate. **(C)** Competitive inhibition assay for IFN-γ assay. Five and ten cells were sorted in 25 μl of lysis buffer and the lysates were incubated with or without anti-IFN-γ inhibition antibody for 45 mins at 4°C. Lysates were transferred to V bottom SiMoA plates and IFN-γ assay was performed. Data are represented as mean and mean ± SD and differences among means were calculated using Mann–Whitney test. *P* < 0.05 were considered significant.

Figure 3A displays the standard curves IFN-γ from experiments performed on three separate days and demonstrates the reproducibility of the SiMoA assay. When sorted singly, CD8+ T cells displayed an average of 6.22 fg IFN-γ per cell and a standard deviation of 2.97 (*n* = 27). Per cell averages of IFN-γ in CD8 T cells showed similar results when sorting 1, 2, 5, or 10 cells per well (6.22, 6.20, 5.93, and 5.44 fg/cell, respectively (*n* = 27), with an overall average of all measurements of 5.95 fg IFN-γ per cell (SD = 0.363, relative SD = 6.11%) (Figure 3B), equal to 185,102 molecules. In order to ascertain the specificity of this assay we performed competitive inhibition assay by pre-incubating single cell lysates with their IFN-γ antibodies. We found significant inhibition of IFN-γ levels in IFN-γ antibodies preincubated wells in comparison to no treatment wells (Figure 3C). These data indicate that sensitivity of SiMoA was sufficient to permit quantification of IFN-γ in individually sorted cells, as adding more cells per lysate (and thus more IFN-γ per lysate) did not drastically affect the per mean cell quantification. When sorted, cells negative for cell surface IFN-γ revealed quantities of intracellular IFN-γ which were below the LLoQ of the SiMoA assay in all but one instance at the single cell level.

### Quantification of TNF-α in single CD8 T cells

**Figure 4:**
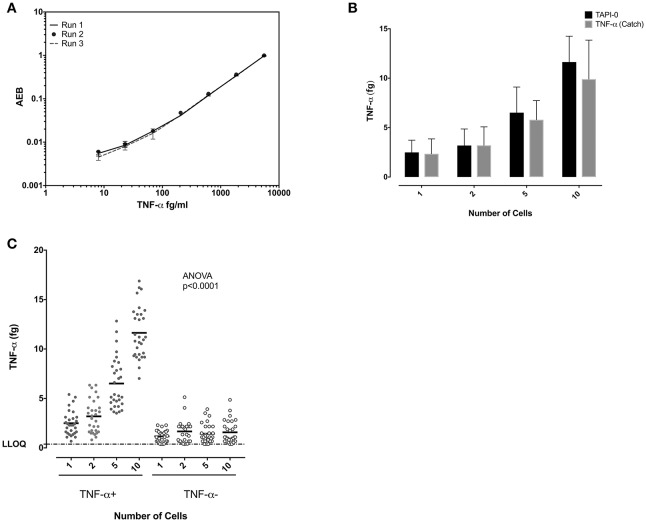
Quantification of TNF-α in single CD8 T cells. **(A)** Reproducibility of the SiMoA standard curves for TNF-α. Standard concentrations of TNF-α were used to generate curve to calculate the unknown concentrations in cells. **(B)** Comparison of TNF-α concentrations using TAPI-0 and bispecific catch reagent assays. The assays were found to yield similar results. **(C)** Comparison of measured TNF-α protein at 1, 2, 5, and10 cell levels in CD8 T cells. The amount of TNF-α measured in femtograms of lysates from TNF-α -producing cells or cells not producing TNF-α. Horizontal axis is number of cells sorted into each lysate. Left are data from TNF-α-surface positive cells, right are data from TNF-α-cell surface negative cells. Data are represented as mean and mean ± SD and differences among means were calculated using one-way ANOVA with Dunnett's multiple comparisons post-test. *P* < 0.05 were considered significant.

Similar experiments were performed to study TNF-α-producing CD8+ T cells. Standard curves of TNF-α from experiments performed on three separate days demonstrated the reproducibility of the SiMoA assay (Figure 4A). Both the anti-TNF-α catch reagents and the TAPI-0 approaches for detecting cytokine secreting cells yielded similar results (Figure 4B). TNF-α-producing CD8+ T cells individually sorted cells showed a mean of 2.49 fg TNF-α per cell (*n* = 30) (SD = 1.23), while sorting 1, 2, 5, and 10 cells per well-lysate had means of 2.49, 1.59, 1.30, and 1.16 fg/cell with an overall average of all measurements of 1.64 fg TNF-α per cell (SD = 0.59, relative SD = 36.5%) (*n* = 30) (Figure 4C) equal to 38,467 molecules. TNF-α-negative cells showed a mean on 0.6 fg/cells (SD = 0.47) (*n* = 30).

### SiMoA assay for comparison of median fluorescent intensities with protein concentration

**Figure 5:**
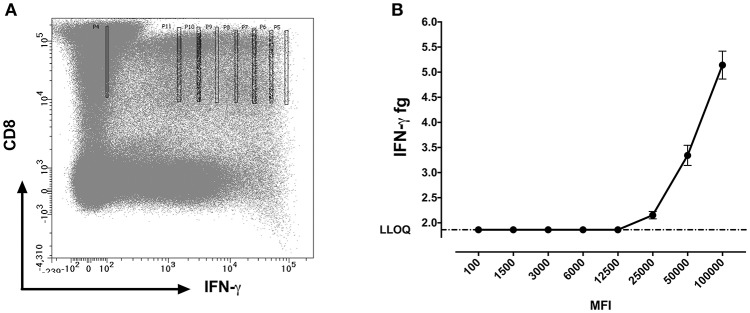
IFN-γ protein quantification at different mean fluorescent intensities (MFI). **(A)** Flow cytometry dot plot showing gating strategy for sorting CD8+IFN-γ+ cells at different MFI's using bin gating. The gates were set as such that the MFI values fall in a range of 100,000 with 8 different intensities following a two-fold incremental order and covering from the brightest to the dimmest IFN-γ expression on CD8 cells **(B)** Measured amounts of IFN-γ from cells sorted from various levels of staining for cells surface IFN-γ expressed as mean ± SD fluorescence intensity channel numbers (MFI).

We further questioned whether the protein concentrations quantified using SiMoA correspond to the mean fluorescence intensities (MFI) of cytokines observed by flow cytometry. MFI is often used in flow cytometry to denote the average relative amount of a molecule as detected by fluorescence in a specific channel. Experiments were performed with 10 cells sorted per well. For MFI values above 12,500, a strong positive correlation (*p* < 0.0001; *R*^2^ = 0.97) was detected between the qualitative MFI measurements and the quantitative SiMoA values of IFN-γ concentrations in CD8 T cells (Figures 5A,B).

## Discussion

Single cell assays have emerged as an important tool in studying cellular heterogeneity. In this study, we report the ultrasensitive detection of pro-inflammatory cytokines IFN-γ and TNF-α in single CD8 T cells in human peripheral blood using only commercially-available instrumentation. Using the power of flow cytometry cell sorting together with an automated SiMoA platform, we demonstrated protein quantification from single human primary cells that can be easily performed without involving PCR, custom made microfluidics, or complex modeling methodologies.

Studies to determine quantitative protein amounts in single cells have been limited to date. A recent study by Rodero et.al (26) quantified IFN-α in healthy *ex-vivo* CD8 T cells and found the amount to be approximately 14 fg/cell. In that study, the cells collected were in a large pool of sorted CD8 cells (~50,000 to 100,000 cells) and the per cell values calculated accordingly. Thus, while Rodero reported a per cells values for this cytokine, no true single cell analyses were performed and therefore the values represent an average of all values and may not reflect the heterogeneity of the entire sample studied. Another study where protein estimation has been performed in a single cell is by Schubert et al. (17) where quantification of prostate specific antigen (PSA) in a single prostate cancer cell was reported. The study was performed by serially diluting cells to achieve single cells, although there was no verification that single cells were collected. Furthermore, that study examined a cell line, not primary cells. Ma and colleagues published a study using fabricated microchips to analyze multiplex analytes from individual cells (27). Our current study differs from that by Ma in two important aspects: (1) Ma et al. measured secreted analytes while in the current study we quantified intracellular analytes, and (2) Ma et al. used a custom fabricated microfluidic chip to analyze single cells while the current study used only commercially available devices that are common at large research centers. While these are significant differences in these studies, it is our feeling that the two studies complement each other and provide differing venues to study cytokines on a single cell basis.

Our assay employs a simple cytokine capture technique which can be performed by using readily available bi-phenotypic monoclonal antibodies against cytokines or as in case of TNF-α using enzyme inhibitor which arrests the surface release of this identifying cytokine-producing cells. SiMoA data quantifying TNF-α in single cells was confirmed in different assays varying in their method of cytokine capture. Interestingly these assays yield similar results for TNF-α concentrations at single cell level which confirms the robustness of these assays and validates the protein concentration observed for single cells. Presumably, if specific cytokine-producing cells could be identified using cell surface makers or tetramer binding, then it would be possible to use those markers to sort cells for use with our current method. There are caveats to our current study. First, as we were sorting cells directly into lysis buffer, we could not directly verify that only one cell per well had been sorted. In a separate experiment, sorting fresh human PBMCs labeled with carboxyfluorescein succinimidyl ester (CFSE) and CD8, we were able to visually verify that single cells had been placed into 190 of the 192 well targeted, 99% accuracy). We therefore believe that a similar degree of accuracy applies to the experimental data. A second caveat is that the values obtained for the cytokine negative samples were at the extreme low end of our standard curves, essentially at the lower levels of quantification, and therefore the accuracy of these is problematic. The ability to quantify proteins at the single cell level opens many exciting applications. Flow cytometry, which is extremely good at detecting low levels of proteins such as cytokines is not truly quantitative; rather results are reported as “bright” or “dim,” or at best as a relative fluorescence index (MFI). Applying the current method to these studies would permit precise quantification and might help to widely standardize such assays. As our assay provides for direct quantification of intracellular analytes, rather than of mRNA, it provides what is likely to be a more relevant biological measurement without any bias introduced by enzymatic amplification. Molecular mechanisms, pathways, and cellular heterogeneity at the single cell level can be studied to potentially enable early disease detection or responses to targeted therapy. Also, this technique will be highly efficient in protein quantification with disease conditions where cell number is a limitation in studying effector function. In conclusion, the current work describes an exquisitely sensitive, robust, and readily available system for the quantification of protein molecules in single cells. This technique provides an important new tool for the field of single cell analysis.

## Ethics statement

This study was carried out in accordance with National Heart Lung and Blood Institute (NHLBI) Institutional Review Board in accordance with the Declaration of Helsinki. All study participants provided written informed consent (protocol-07-H-0113).

## Author contributions

AS performed experiments, analyzed data, and assisted in writing the manuscript. PD and AD assisted in performing experiments. JM conceived this study, assisted analyzing the data, and wrote the manuscript.

### Conflict of interest statement

The authors declare that the research was conducted in the absence of any commercial or financial relationships that could be construed as a potential conflict of interest.

## Supplementary material

Supplementary Figure 1 Sample CV profile of serially diluted samples. **(A)**. IFN-γ and **(B)**. TNF-α. Concentration (x-axis) vs. CV% (y-axis) with power fit; LLoQ concentration is calculated by solving the power fit equation when y = 20%.
