# Supplementary figures and images for: Ultrasensitive Quantification of Cytokine Proteins in Single Lymphocytes From Human Blood Following ex-vivo Stimulation
Source: Front Immunol. 2018 Oct 23;9:2462. doi: 10.3389/fimmu.2018.02462 (PMC6206239; doi:10.3389/fimmu.2018.02462)

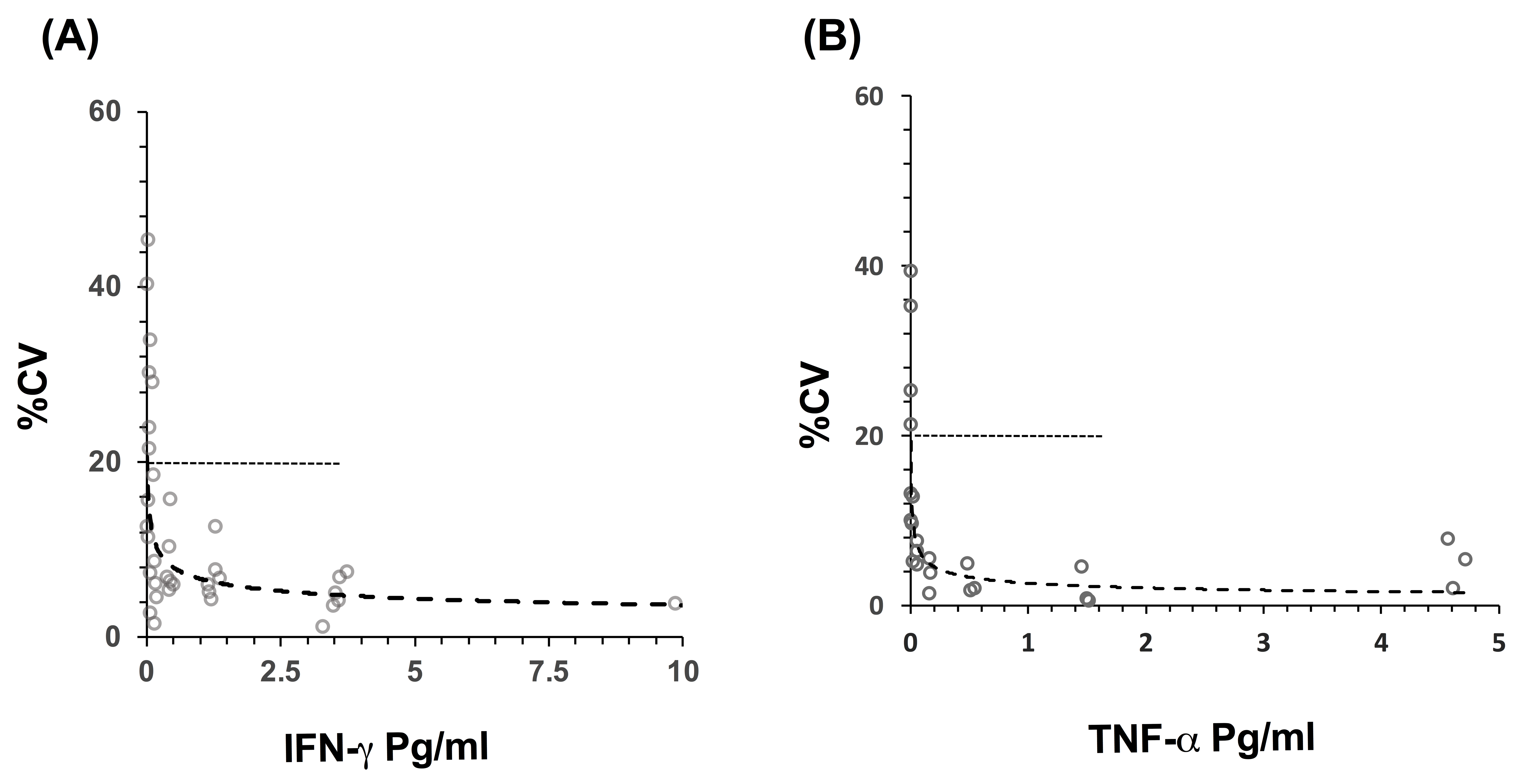

Supplement: Supplementary Figure 1 — Sample CV profile of serially diluted samples. (A). IFN-γ and (B). TNF-α. Concentration (x-axis) vs. CV% (y-axis) with power fit; LLoQ concentration is calculated by solving the power fit equation when y = 20%. [file Image_1.TIFF]
